# Synthesis and psychobiological evaluation of modafinil analogs in mice

**DOI:** 10.1186/2008-2231-21-67

**Published:** 2013-12-19

**Authors:** Arezou Lari, Isaac Karimi, Hadi Adibi, Alireza Aliabadi, Loghman Firoozpour, Alireza Foroumadi

**Affiliations:** 1 Students Research Committee, Faculty of Pharmacy, Kermanshah University of Medical Sciences, Kermanshah, Iran; 2 Laboratory of Molecular and Cellular Biology, School of Veterinary Medicine, Razi University, Kermanshah, Iran; 3 Novel Drug Delivery Research Center, Faculty of Pharmacy, Kermanshah University of Medical Sciences, Kermanshah, Iran; 4 Drug Design and Development Research Center, Tehran University of Medical Sciences, Tehran, Iran; 5 Neuroscience Research Center, Institute of Neuropharmacology, Kerman University of Medical Sciences, Kerman, Iran

**Keywords:** Modafinil, Wake-promoting agent, Narcolepsy

## Abstract

**Background and the purpose of the study:**

Modafinil, a novel wake-promoting agent with low potential for abuse and dependence, has a reliable structure to find some novel derivatives with better activity and lower potential for abuse and risk of dependency. This study was designed to evaluate psychobiological activity of some novel *N*-aryl modafinil derivatives.

**Methods:**

Seven novel *N*-aryl modafinil derivatives were synthesized through three reactions: a) preparation of benzhydrylsulfanyl acetic acid through reaction of benzhydrol with thioglycolic acid, b) formation of desired amide by adding the substituted aniline to activated acid with EDC (1-ethyl-3-(3-dimethyl amino propyl) carbodiimide). This reaction was catalyzed by HOBt (*N*- hydroxylbenzotriazole), and c) oxidation of sulfur to sulfoxide group with H_2_O_2_. Then, their psychobiological effect on the performance of male albino mice were compared to that of modafinil as following: wakefulness by determining the effects of derivatives on phenobarbital-induced loss of the righting reflex (LOPR); exploratory activity by measuring activity in the open field test (OFT); depression by measuring immobility time (IT) during forced swimming test (FST) and the anxiogenic and anxiolytic like effects by using elevated plus-maze test (EPM). All tests were videotaped and analyzed for the frequency and duration of the behaviors during the procedures.

**Conclusions:**

2-(Benzhydrylsulfonyl)-*N*-(4-chlorophenyl)acetamide (**4c**) showed comparable result in LOPR test. However, all analogs were found to be stimulant except 2-(benzhydrylsulfinyl)-*N*-phenylacetamide (**4a**). Also **4c** led the most exploratory activity in mice among derivatives. FST results showed that 4a had the longest IT while modafinil, 2-(benzhydrylsulfinyl)-*N*-(3-chlorophenyl) acetamide (**4b**) and 2-(benzhydrylsulfinyl)-*N*-(4-ethylphenyl) acetamide (**4d**) had the shortest IT. In EPM, all derivatives showed anxiogenic-like behavior since they decreased open arms time and open arms entries and simultaneously increased close arms time.

## Introduction

Narcolepsy is a neurological sleep disorder that is estimated to affect as many as 200,000. It is as widespread as multiple sclerosis and more prevalent than cystic fibrosis, but it is less well known [1,2].

**Figure 1:**
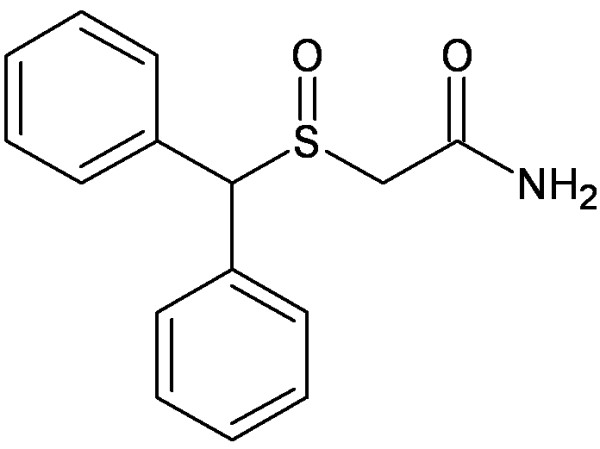
Chemical structure of modafinil.

The main treatment of narcolepsy is using of central nervous system (CNS) stimulants such as amphetamine, methylphenidate and modafinil which is widely regarded as the first-line medication for narcolepsy (Figure 1) [1,3]. Amphetamine and methylphenidate are associated with a significant abuse potential while modafinil which has lower abuse potential [4,5]. Surprisingly, modafinil is used sometimes to treat methamphetamine dependency; however this type of therapy has not been authoritized [6].

**Scheme 1:**
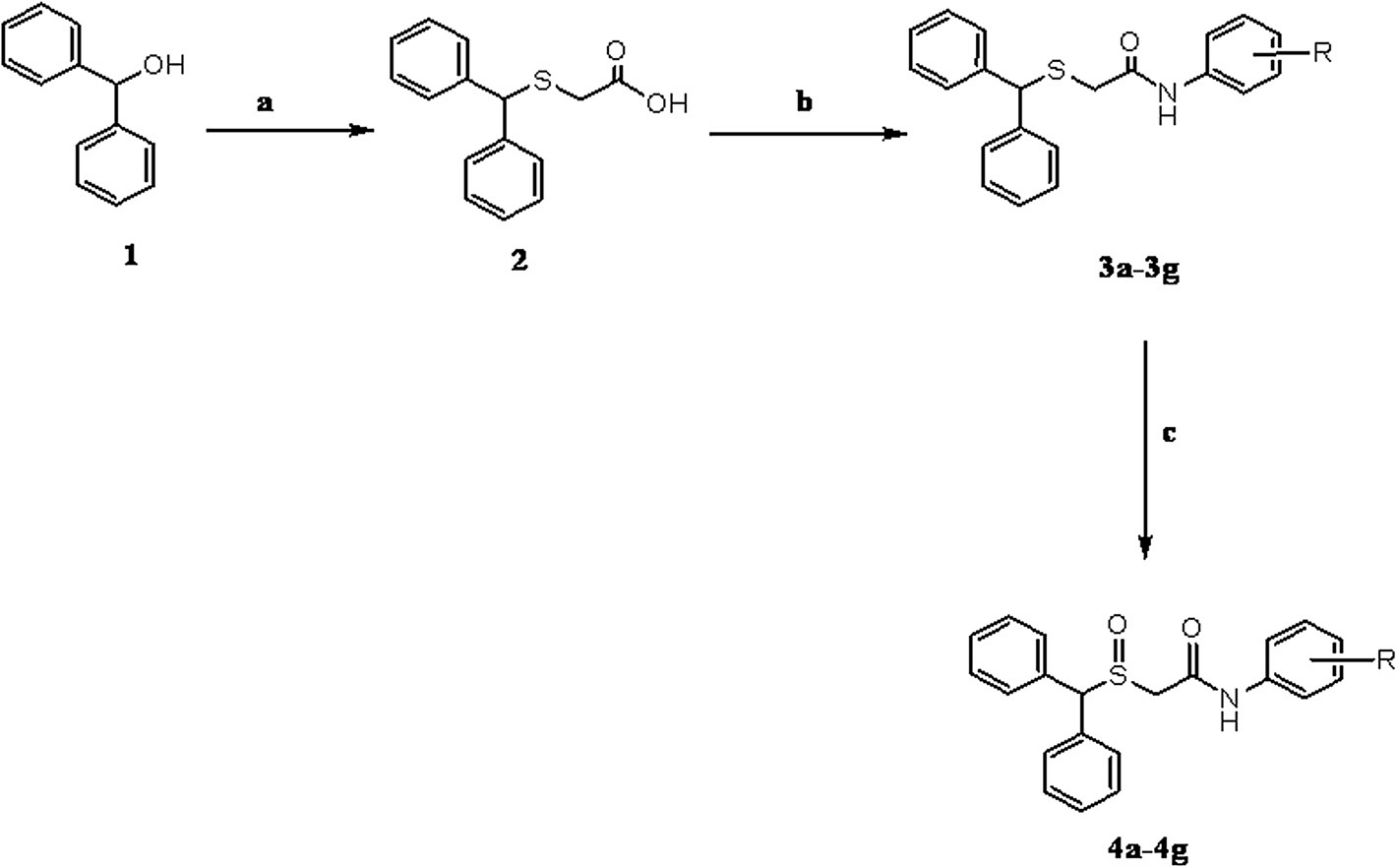
**Synthesis of target compounds 4a-4g (R: (a) = H, (b) = 3-Cl, (c) = 4-Cl, (d) = 4-Et, (e) = 3,4-Cl, (f) = 4-NO**_**2**_**, (g) = 4-Br), Reagents and conditions: (a) Thioglycolic acid, TFA, 3 h; (b) appropriate amine, EDC, HOBt; (c) appropriate amide, H**_**2**_**O**_**2**_**, acetic acid.** **Synthesis of target compounds 4a-4g (R: (a) = H, (b) = 3-Cl, (c) = 4-Cl, (d) = 4-Et, (e) = 3,4-Cl, (f) = 4-NO**_**2**_**, (g) = 4-Br), Reagents and conditions: (a) Thioglycolic acid, TFA, 3 h; (b) appropriate amine, EDC, HOBt; (c) appropriate amide, H**_**2**_**O**_**2**_**, acetic acid.**

In one study, some modafinil analogs were evaluated for their CNS activity [7]. Most of the derivatives of nitrogen group like NHCH_3_, NHCH (CH_3_)_2_, HCN (CH_3_)_3_ were stimulant, although some analogs with piperidine or morpholine groups were sedative. Here, synthesis and psychobiological evaluation of novel modafinil derivatives with different *N-Aryl* moieties were reported. These analogs with suitable Log *P* were chosen due to their easy transfer across the blood brain barrier. The tilted compounds were prepared according to Scheme 1. The key intermediate 2-(benzhydrylthio) acetic acid (**2**) was prepared from benzhydrol and thioglycolic acid. Amidation of appropriate aniline with 2-(benzhydrylthio)acetic acid yielded the corresponding amide (**3a-3g**). The obtained amides (**3a-3g**) gently oxidized by H_2_O_2_ to form the corresponding sulfoxide derivatives (**4a-4g).**

## Material and methods

### Chemistry

All chemical reagents and solvents used in this study were purchased from Merck AG (Darmstadt, Germany). Melting points were determined by Kofler hot stage apparatus and are not corrected. The IR spectra were obtained on a Shimadzu 470 spectrophotometer (potassium bromide disks). NMR spectra were appropriately recorded using a Bruker 400 spectrometer (Bruker Bioscience, Billerica, MA, USA), and chemical shifts were expressed as δ (ppm) with tetramethylsilane as internal standard. The mass spectra were run on a Finigan TSQ-70 spectrometer (Finigan, USA) at 70 eV. Merck silica gel 60 F_254_ plates were used for analytical TLC.

#### Synthesis of 2-(benzhydrylthio)acetic acid (2)

A mixture of benzhydrol (50.0 g, 271.4 mmol) and thioglycolic acid (25.0 g, 271.4 mmol) in trifluoroacetic acid (300 mL) was stirred at room temperature for 3 h. The solvent was removed under reduced pressure to afford a crude solid. Water (300 ml) was added and the resulting precipitate collected by filtration. The solid was washed with *n*-hexane (400 ml) and dried to afford a white solid (69.2 g).

Yield: 99%, mp: 126–129°C. IR (KBr, cm^-1^); ῡ: 3071, 2570, 1941, 1860, 1809, 1689, 1596, 1491, 1301, 1203, 1137, 1209, 1021, 804. ^1^H-NMR (CDCl_3_, 400 MHz) *δ* (ppm): 3.1 + .3699(s, 2H, -SCH_2_CO-), 5.5 (s, 1H, Ph-CH-Ph), 7.13 (t, *J* = 7.6 Hz, aromatic), 7.22-7.25 (m, aromatic), 7.25 (m, aromatic), 7.33 (m, aromatic), 7.45 (m, aromatic) [8].

#### General procedure for the synthesis of compounds 3a-3g

The mixture of 2-(benzhydrylthio) acetic acid **(2)**, EDC (1-ethyl-3-(3-dimethylaminopropyl) carbodiimide) (1 mol) and HOBt (hydroxybenzotriazole) (1 mol) in acetonitrile solvent was kept under stirring for 30 min in order to activate acid group. Afterwards, appropriate aniline derivative was added and the mixture was stirred at room temperature for 24 h. The solvent was evaporated and ethyl acetate was added to the residue. The organic phase was washed with sulfuric acid 5%, sodium bicarbonate and brine. Then, the organic layer was dried over anhydrous sodium sulfate, filtered and evaporated to dryness. The residue was chromatographed on silica gel plate eluting with ethyl acetate/petroleum [9].

#### 2-(benzhydrylthio)-*N*-phenylacetamide (3a)

Yield: 81%, mp: 90°C. IR (KBr, cm^-1^); ῡ: 3431, 3243, 3060, 2959, 2854, 1655, 1597, 1547, 1491, 1443, 1325, 754, 696. ^1^H-NMR (CDCl_3_, 400 MHz) *δ* (ppm): 3.26 (s, 2H, -SCH_2_CO-), 5.18 (s, 1H, Ph-CH-Ph), 7.13 (t, *J* = 7.6 Hz, aromatic), 7.22-7.25 (m, aromatic), 7.30-7.35 (m, aromatic), 7.41 (d, *J* = 8 Hz, aromatic), 7.48 (d, *J* = 8 Hz, aromatic), 8.41 (brs, 1H, NH).

#### 2-(benzhydrylthio)-*N*-(3-chlorophenyl) acetamide (3b)

Yield: 73%, mp: 60°C. IR (KBr, cm^-1^); ῡ: 3426, 3329, 3229, 3064, 2923, 1665, 1641, 1593, 1525, 1421, 1310, 1240, 1129, 1074, 878, 775, 698. ^1^H-NMR (CDCl_3_, 400 MHz) *δ* (ppm): 3.27 (s, 2H, -SCH_2_CO-), 5.18 (s, 1H, PhCHPh), 7.15 (m, aromatic), 7.25-7.41 (m, aromatic), 8.42 (brs, 1H, NH).

#### 2-(benzhydrylthio)-*N*-(4-chlorophenyl) acetamide (3c)

Yield: 78%, mp: 90°C. IR (KBr, cm^-1^); ῡ: 3430, 3121, 3067, 2923, 2853, 1646, 1549, 1490, 1400, 1093, 1034, 823, 700. ^1^H-NMR (CDCl_3_, 400 MHz) *δ* (ppm): 3.27 (s, 2H, -SCH_2_CO-), 5.15 (s, 1H, PhCHPh), 7.25-7.41 (m, 14H, aromatic), 8.41 (s, 1H, NH).

#### 2-(benzhydrylthio)-*N*-(4-ethylphenyl) acetamide (3d)

Yield: 72%, mp: 87°C. IR (KBr, cm^-1^); ῡ: 3258, 3060, 2962, 2925, 2873, 1638, 1599, 1535, 1449, 1410, 1321, 1124, 1073, 1025, 971, 825, 743, 697. ^1^H-NMR (CDCl_3_, 400 MHz) *δ* (ppm): 1.22 (t, 3H, CH_3_), 2.62 (q, 2H, CH_2_), 3.25 (s, 2H, -SCH_2_CO-), 5.19 (s, 1H, PhCHPh), 7.15-7.42 (m, 14H, aromatic), 8.40 (s, 1H, NH).

#### 2-(benzhydrylthio)-*N*-(3, 4-dichlorophenyl) acetamide (3e)

Yield: 75%, mp: 85°C. IR (KBr, cm^-1^); ῡ: 3400, 3268, 3087, 2922, 1710, 1650, 1591, 1528, 1480, 1376, 1318, 1118, 1027, 870, 811, 748, 697. ^1^H-NMR (CDCl_3_, 400 MHz) *δ* (ppm): 3.28 (s, 2H, -SCH_2_CO-), 5.14 (s, 1H, PhCHPh), 7.65 (s, 1H, H_2_-Dichlorophenyl) and 7.24-7.40 (m, 12H, aromatic), 8.37 (brs, 1H, NH).

#### 2-(benzhydrylthio)-*N*-(4-nitrophenyl) acetamide (3f)

Yield: 68%, mp: 80-84°C. IR (KBr, cm^-1^); ῡ: 3313, 3087, 3017, 2923, 1675, 1616, 1596, 1552, 1500, 1334, 1307, 1255, 1117, 853, 748, 696. ^1^H-NMR (CDCl_3_, 400 MHz) *δ* (ppm): 3.34 (s, 2H, -SCH_2_CO-), 5.16 (s, 1H, PhCHPh), 7.24 (d, *J* = 8Hz, aromatic), 7.31 (t, *J* = 4Hz, aromatic), 7.4 (d, *J* = 8Hz, aromatic), 7.61 (d, 2H, *J* = 8Hz, *p*-Nitrophenyl), 8.19 (d, 2H, *J* = 8.8Hz*, p*-Nitrophenyl), 8.64 (brs, 1H, NH).

#### 2-(benzhydrylthio)-*N*-(4-bromophenyl) acetamide (3g)

Yield: 84%, mp: 92°C. IR (KBr, cm^-1^); ῡ: 3237, 3027, 2927, 1714, 1641, 1593, 1533, 1490, 1396, 1314, 1125, 1072, 1008, 818, 746, 696. ^1^H-NMR (CDCl_3_, 400 MHz) *δ* (ppm): 3.28 (s, 2H, -SCH_2_CO-), 5.17 (s, 1H, PhCHPh), 7.32-7.48 (m, 14H, aromatic), 8.42 (brs, 1H, NH).

#### General procedure for the synthesis of 2-(benzhydrylsulfinyl)-*N*-phenylacetamide (4a-4g)

2-(benzhydrylthio)-*N*-phenylacetamide (3.46 g, 0.013 mol) was taken in glacial acetic acid (14 ml) with stirring. 1.34 ml of 30% H_2_O_2_ was added with chilling in ice water. The mixture was left in the refrigerator for 4 h and thereafter worked up by treating with 70 ml of ice-cold water. The precipitated material was filtered under suction and washed with ice-cold water to give 1.5 g of white crystals (43%), mp: 159-160°C [10].

#### 2-(benzhydrylsulfinyl)-*N*-phenyl acetamide (4a)

Yield: 70%, mp: 98°C. IR (KBr, cm^-1^); ῡ: 3426, 3056, 2924, 2854, 1673, 1600, 1551, 1493, 1446, 1325, 1112, 1033, 755, 698.

^1^H-NMR (d ppm, CDCl_3_, 400 MHz): 3.23 (d, 1H, -SCH_2_CO-, *J* = 12Hz), 3.66 (d, 1H, -SCH_2_CO-, *J* = 12Hz), 5.25 (s, 1H, PhCHPh), 7.12 (t, *J* = 8Hz, aromatic), 7.26 (s, 1H, aromatic), 7.31 (t, *J* = 8Hz, aromatic), 7.36-7.52 (m, aromatic), 9.21 (brs, 1H, NH). ^13^C-NMR (125 MHz, CDCl_3_): *δ* 51.95 (S-CH_2_), 71.54 (S-CH), 120.15 (C_2, 6_ aniline), 124.64 (C_4_ aniline), 128.77 (C_3, 5_ aniline), 128.87 (C_3,5_ phenyl), 128.97 (C_4_ phenyl), 129.51 (C_2,6_ phenyl), 133.78 (C_1_ phenyl), 134.29 (C_1_ aniline), 175.00 (C = O). MS (m/z): 349 (M^+^), 309, 167, 119, 104, 93, 77, 65, 57, 43.

#### 2-(benzhydrylsulfinyl)-*N*-(3-chlorophenyl) acetamide (4b)

Yield: 68%, mp: 160°C. IR (KBr, cm^-1^); ῡ: 3441, 3250, 3184, 3066, 3026, 2923, 2856, 1682, 1596, 1546, 1480, 1430, 1372, 1320, 1035, 794, 700. ^1^H-NMR (CDCl_3_, 400 MHz) *δ* (ppm): 3.23 (d, 1H, -SCH_2_CO-, *J* = 12Hz), 3.67 (d, 1H, -SCH_2_CO-, *J* = 12Hz), 5.27 (s, 1H, PhCHPh), 7.08-7.50 (m, 13H, aromatic), 7.69 (s, 1H, H_2_-*m*-Chlorophenyl), 9.35 (brs, 1H, NH). ^13^C-NMR (125 MHz, CDCl_3_): *δ* 52.34 (S-CH_2_), 71.38 (S-CH), 117.88 (C_3_ aniline), 120.04 (C_2_ aniline), 124.55 (C_4_ aniline), 128.84 (C_3, 5_ phenyl), 128.90 (C_4_ phenyl), 128.99 (C_2,6_ phenyl), 129.51 (C_6_ aniline), 131.94 (C_5_ aniline), 133.67 (C_1_ phenyl), 134.37 (C_1_ aniline), 162.16 (C = O). MS (m/z): 385 (M^+^+2), 383 (M^+^), 293, 201, 167, 153, 127, 111, 91, 64, 47.

#### 2-(benzhydrylsulfinyl)-*N*-(4-chlorophenyl) acetamide (4c)

Yield: 73%, mp: 170°C. IR (KBr, cm^-1^); ῡ: 3444, 3248, 2920, 1684, 1597, 1541, 1489, 1398, 1320, 1246, 1037, 743, 701. ^1^H-NMR (CDCl_3_, 400 MHz) *δ* (ppm): 3.26 (d, 1H, -SCH_2_CO-, *J* = 16Hz), 3.66 (d, 1H, -SCH_2_CO-, *J* = 16Hz), 5.26 (s, 1H, PhCHPh), 7.24 (d, 2H, *J* = 8Hz, *p*-Chlorophenyl), 725–7.50 (m, 12H, aromatic), 9.33 (brs, 1H, NH). ^13^C-NMR (125 MHz, CDCl_3_): *δ* 52.21 (S-CH_2_), 71.52 (S-CH), 121.19 (C-Cl), 128.85 (C_2, 6_ aniline), 128.91 (C_3,5_ phenyl), 128.98 (C_4_ phenyl), 129.52 (C_2,6_ phenyl), 129.56 (C_3,5_ aniline), 134.29 (C_1_ phenyl), 136.18 (C_1_ aniline), 162.08 (C = O). MS (m/z): 385 (M^+^+2), 383 (M^+^), 167, 153, 127, 111.

#### 2-(benzhydrylsulfinyl)-*N*-(4-ethylphenyl) acetamide (4d)

Yield: 74%, mp: 158°C. IR (KBr, cm^-1^) ῡ: 3253, 3185, 3058, 2957, 2923, 2858, 1679, 1540, 1412, 1322, 1043, 957, 832, 747, 701.

^1^H NMR (CDCl_3_, 400 MHz) *δ* (ppm): 1.23 (t, 3H, CH_3_), 2.63 (q, 2H, CH_2_), 3.23 (d, 1H, *J* = 12Hz, -SCH_2_CO-), 3.66 (d, 1H, -SCH_2_CO-, *J* = 12Hz), 5.21 (s, 1H, PhCHPh), 7.16-7.48 (m, 14H, aromatic), 9.21 (brs, 1H, NH). ^13^C-NMR (125 MHz, CDCl_3_): *δ* 15.66 (CH_3_), 28.33 (CH_2_), 36.97 (S-CH_2_), 55.09 (S-CH), 120.22 (C_4_ aniline), 127.53 (C_2, 6_ aniline), 128.25 (C_3,5_ phenyl), 128.34 (C_4_ phenyl), 128.46 (C_2,6_ phenyl), 128.84 (C_3,5_ aniline), 135.02 (C_1_ phenyl), 140.10 (C_1_ aniline), 166.28 (C = O). MS (m/z): 377 (M^+^), 284, 279, 191, 167, 149, 105, 85, 71, 57.

#### 2-(benzhydrylsulfinyl)-*N*-(3, 4-dichlorophenyl) acetamide (4e)

Yield: 63%, mp: 140°C. IR (KBr, cm^-1^); ῡ: 3293, 3258, 3101, 3052, 2912, 1711, 1686, 1587, 1383, 1312, 1224, 1146, 1036, 878, 820, 742, 698. ^1^H NMR (CDCl_3_, 400 MHz) *δ* (ppm): 3.24 (d, 1H, *J* =16Hz, -SCH_2_CO-), 3.67 (d, 1H, *J* = 16Hz, -SCH_2_CO-), 5.32 (s, 1H, PhCHPh), 7.23-7.48 (m, 12H, aromatic), 7.77 (s, 1H, H_2_-*m*-Chlorophenyl), 9.45 (brs, 1H, NH). ^13^C-NMR (125 MHz, CDCl_3_): *δ* 52.13 (S-CH_2_), 71.41 (S-CH), 118.99 (C_4_ aniline), 121.48 (C_2_ aniline), 121.56 (C_6_ aniline), 128.87 (C_3,5_ phenyl), 129.04 (C_4_ phenyl), 129.50 (C_2, 6_ phenyl), 129.56 (C_3_ aniline), 131.94 (C_5_ aniline), 133.64 (C_1_ phenyl), 134.19 (C_1_ aniline), 162.14 (C = O). MS (m/z): 421 (M^+^4), 419 (M^+^+2), 417 (M^+^), 199, 184, 167, 149, 105.

#### 2-(benzhydrylsulfinyl)-*N*-(4-nitrophenyl) acetamide (4f)

Yield: 76%, mp: 198°C. IR (KBr, cm^-1^); ῡ: 3448, 3202, 3078, 2922, 2852, 1702, 1618, 1598, 1566, 1497, 1335, 1251, 1159, 1039, 859, 748, 698. ^1^H NMR (CDCl_3_, 400 MHz) *δ* (ppm): 3.47 (d, 1H, -SCH_2_CO-, *J* = 12Hz), 3.76 (d, 1H, -SCH_2_CO-, *J* = 12Hz), 5.42 (s, 1H, PhCHPh), 7.35-7.41 (m, 8H, aromatic), 7.53 (d, 2H, *J* = 8.4Hz, aromatic), 7.78(d, 2H, *J* = 8Hz, *p*-Nitrophenyl), 8.16 (d, 2H, *J* = 8Hz, *p*-Nitrophenyl), 10.40 (brs, 1H, NH). MS (m/z): 394 (M^+^), 279, 257, 236, 167, 149, 69, 57, 43.

#### 2-(benzhydrylsulfinyl)-*N*-(4-bromophenyl) acetamide (4g)

Yield: 66%, mp: 155°C. IR (KBr, cm^-1^); ῡ: 3430, 2923, 2853, 1741, 1663, 1630, 1454, 1379, 1240, 1155, 1034, 837, 743, 700. ^1^H NMR (CDCl_3_, 400 MHz) *δ* (ppm): 3.24 (d, 1H, -SCH_2_CO-, *J* = 16Hz), 3.65 (d, 1H, -SCH_2_CO-, *J* = 16Hz), 5.33 (s, 1H, PhCHPh), 7.26-7.49 (m, 14H, aromatic), 9.44 (s, 1H, NH). ^13^C-NMR (125 MHz, CDCl_3_): δ 51.68 (S-CH_2_), 71.76 (S-CH), 117.24 (C-Br), 121.82 (C_2, 6_ aniline), 128.82 (C_3,5_ phenyl), 129.00 (C_4_ phenyl), 129.43 (C_2, 6_ phenyl), 129.55 (C_3, 5_ aniline), 131.94 (C_1_ phenyl), 136.61 (C_1_ aniline), 162.19 (C = O). MS (m/z): 429 (M^+^+2), 428 (M^+^+1), 368, 362, 167, 152, 69, 57, 43.

### Psychobiological activity

#### Animals

This study approved by the Laboratory Animal Care Committee of School of Veterinary Medicine, Razi University, Kermanshah, Iran. The experiments were carried out on male albino mice weighing 20–25 g at the beginning of the experiments. The animals were maintained under standard laboratory conditions (12-h light/dark cycle, room temperature 21 ± 1°C) with free access to tap water and laboratory chow (Dan-e-pars Co., Kermanshah, Iran) except during brief periods of experiments, and were adapted to the laboratory conditions for at least 1 week. Each experimental group consisted of 5–6 animals.

#### Drugs

The compounds tested were: modafinil (Modiodal®, Cephalon, France), Phenobarbital (Chemi darou product, Iran) and our made modafinil derivatives. All agents were diluted to an adequate concentration using dimethyl sulfoxide (DMSO). They were administered intraperitoneally (i.p.) 30 min prior to each behavioral test. Control groups received DMSO injection at the same volume and by the same route. The doses of modafinil and its derivatives (100 mg/kg) employed in the present study was adopted from previous study [11]. The dose of phenobarbital (50 mg/kg) was chosen according to that commonly used dose reported in the literature [12]. In all tests, each mouse was tested once.

#### Phenobarbital-induced loss of righting reflex

Phenobarbital (50 mg/kg i.p.) was administered to each mouse. The loss of righting reflex (LORR) was measured as the time interval between losing and recovery of the righting reflex after phenobarbital administration. Recovery of the righting reflex was defined as the ability of the animal to return to its feet 3 times within 60 sec when placed on its back [13]. Mice received modafinil or its derivatives (100 mg/kg, i.p.) 30 min before i.p. injection of phenobarbital. The ethological room was illuminated with a soft light and external noise was attenuated.

#### Open field test (OFT)

The open field consisted of a square arena (60 × 60 cm^2^), with a white floor divided into 36 squares (10 × 10 cm^2^), enclosed by continuous, 25-cm-high walls made of glass. The test was initiated by placing a single mouse in the middle of the arena and letting him move freely for 5 min. The mice had not been pre-exposed to the arena. Mouse behavior was continuously videotaped by a video camera placed over the apparatus and the arena was carefully cleaned with alcohol and rinsed with water after every test to eliminate olfactory cues. Decrease of the latency to enter the central part was considered as an indicator of anxiolysis and locomotor activity was evaluated by counting the number of segments crossed with a 4-paw as described previously [14].

#### Forced swimming test (FST)

Mice were individually forced to swim in a plastic cylinder (25 × 25 × 40 cm^3^) containing 18 cm of water at 22°C. This volume of water precluded mice touching the bottom with their feet or tails. Mice were submitted to the procedure for 15 min on the first day (pretest) and for 5 min on the second day test, 24 h later. Each mouse i.p. received the tested derivatives 30 min before forced swimming paradigm. At the end of the swimming exposition, the animals were removed from the water and gently dried. The initial 5 min of both swimming sessions were videotaped for behavioral analysis. The immobility time (IT) was recorded only during the last 4 min of these periods, and was defined as the sum of time that the animal was floating, with the face above the water surface and making only slight movements with the front paws to keep from submerging as described previously [15]. The frequency of alternation between mobility and immobility behaviors gradually decreased as time lapsed, the animals tending to remain much more immobile. The decrease and increase of immobility time were interpreted as antidepressive or depressive actions, respectively [16].

#### Elevated plus maze (EPM)

Behavioral effect of modafinil and its derivatives were elevated in the mouse EPM paradigm. The experimental apparatus is shaped like a “plus” sign and consists of a central platform (5 × 5 cm), two open arms (30 × 15 × 5 cm) and two equal-sized closed arms opposite to each other. The maze is made of wood, elevated to a height of 50 cm above the floor. A video camera was mounted vertically about 1meter above the plus-maze for recording behavioral responses. The test consisted of placing a mouse in the central platform facing an enclosed arm and allowed it to freely explore the maze for 5 min. Entry into one arm was defined as the animal placing all four paws into that arm. The test arena was wiped with a damp cloth after each trial. The number of entries into the open and closed arms and the time spent in open arms were measured in the offline condition. Anxiolytic activity was indicated with increase of time spent in open arms or with number of open arms entries while anxiogenic effects are characterized with decrease of these measures.

For the purpose of analysis, open-arm activity was quantified as the amount of time that the rat spent in the open arms relative to the total amount of time spent in open arm (open/total × 100), and the number of entries into the open arms was quantified relative to the total number of entries into open arm (open/total × 100) [17].

#### Statistics

The data are expressed as mean ± S.E.M. The statistical analyses were performed using one-way analyses of variance (ANOVA). Post-hoc comparison of means was carried out with the Tukey's test for multiple comparisons, when appropriate. All data were analyzed using the General Linear Models Procedure of SPSS ver.16 (SPSS Inc., Chicago, IL, USA). The confidence limit of P <0.05 was considered as statistically significant.

## Results and discussion

### Chemistry

Our synthetic route to target compounds **4a-4g** (Table 1) is shown in Scheme 1. The key intermediate 2-(benzhydrylthio) acetic acid **2** was prepared from benzhydrol and thioglycolic acid in trifluoroacetic acid (TFA). 1-Ethyl-3-(3-dimethylaminopropyl) carbodiimide (EDC) was treated with 2-(benzhydrylthio) acetic acid **2** in the presence of hydroxybenzotriazole (HOBt) and stirred for 30 min in acetonitrile. Then, appropriate amine derivative was added and stirring was continued overnight. Thereafter the mixture was washed sequentially with %5 NaHCO_3_ and saturated NaCl solutions, and then dried over Na_2_SO_4_. Removal of the solvent under reduced pressure afforded the amide derivatives **3a-3g**. The obtained amide derivatives **3a-3g** were gently oxidized by H_2_O_2_ to form the corresponding sulfoxide derivatives **4a-4g** (Table 1) and analyzed by ^1^H NMR, infrared, mass spectroscopy and melting point.

**Table 1:** **Effects of *N*-aryl derivatives of modafinil on behavior of albino mice in the elevated plus-maze**

**Compound**	**Open arms entries (O.E)**	**Open arms time spend (O.T)**	**Close arms entries (C.E)**	**Close arms time spend (C.T)**
Modafinil	8.25(2.17)	71.7(10.5)	11.75(3.88)	228.2(10.5)
**4a**	1.00(1.00)^a^	1.5(1.5)^aab^	8.00(3.82)	221.2(23.8)^aa^
**4b**	7.25(1.25)	84.5(19.3)	6.25(0.47)	215.5(19.3)
**4c**	0.00(0.00)^aab^	0.0(0.0)^aab^	2.00(0.00)	285.0(6.4)^aa^
**4d**	3.50(1.50)	57.5(17.5)	4.50(1.50)	242.5(17.5)
**4e**	0.00(0.00)^a^	0.0(0.0)^aa^	1.50(0.50)	300.0(0.0)^aa^
**4f**	6.00(1.00)	120.0(10.0)	6.50(1.50)	180.0(10.0)
**4g**	2.50(1.190) ^a^	16.75(10.0)^aa^	8.25(2.59)	216.2(26.7)^aa^
Control	12.25(2.86)	123.5(19.5)	10.50(3.92)	175.5(19.1)

Data are presented as mean ± S.E.M.

^a^P < 0.05 *vs.* control group, ^aa^P < 0.001 *vs.* control group, ^b^P < 0.05 *vs.* modafinil group.

^1^H NMR spectrum of intermediate **2** showed the benzylic hydrogen was more deshielded (5.50 ppm) than benzylic hydrogen of benzhydrol. Based on ^1^H NMR analysis of intermediates **3a-3g**, the corresponding signals of protons CH benzylic, methylene group adjacent to carbonyl substituent, and NH were appeared within 5.0-5.2 ppm as a singlet, 3.2-3.5 ppm as a singlet, 8.40-8.65 ppm as a broad singlet respectively. Broad singlet peak of the NH proton is a good sign for formation of the amidic bond in this series. The aromatic hydrogens of the phenyl rings are generally appeared in the range between 7.0-8.0 ppm. The ^1^H NMR spectra of compounds **4a-4g** corresponding to the methylene group between carbonyl and sulfoxide substituents showed a doublet of doublet splitting pattern. This behavior is due to existence of two diastereotopic hydrogens of the methylene group. In addition, mass spectrometry analysis of the synthesized compounds **4a-4g** showed expectable fragmentation and hence established the structure of modafinil derivatives. Potassium bromide (KBr) disk was used to obtain the infra red (IR) spectrum related to each compound. The peak related to the carbonyl group appeared in <1700 cm^-1^ is a sign of the amidic carbonyl group in IR spectrum and it is a confirmation for the formation of the amidic moiety in these compounds.

### Psychobiological activity

#### LORR

For comparability of data, we had to use same doses of derivatives. Based on previous study [10], 100 mg/kg of modafinil could be a suitable dose. In this dose, some analogs like **4c** made mice subconscious after i.p. administration of phenobarbital. On the other hand, some mice which received compound **4a**, died. This may be due to more sedative activity of this analog.

In comparison with control animals, compound **4a** significantly increased phenobarbital-induced LORR while other compounds demonstrated a decrease in the duration of LORR.

The experiment showed that compound **4c** was the best CNS stimulant among our synthetic derivatives however it was a slightly weaker than modafinil. Stimulant activity of used compounds is ranged in the following order:

Modafinil > **4c** > **4g** > **4d** > **4f** > **4b** > **4e ** >  DMSO (control) > **4a**

#### OFT

**Figure 2:**
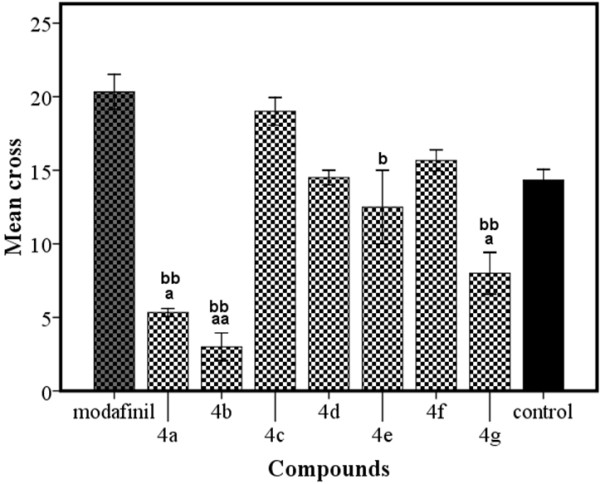
**Exploratory activity (mean ± S.E.M.) in albino mice in the open field test.**^a^P < 0.05, ^aa^P < .001 *vs.* control and ^b^P < 0.05, ^bb^P < 0.001 *vs.* modafinil groups.

The results of the duration of active exploration in the OFT are presented in Figure 2. Mean square crossing of compound **4c**, **4d** and **4f** did not show significant differences when compared to the respective control and modafinil groups. Compound **4a**, **4b** and **4g** were recognized to significantly reduce square crossing compared to control (**4a**, **4g**: p < 0.05, **4b**: p < 0.001). Furthermore, the square crossing of compounds **4a**, **4b**, **4e** and **4g** were significantly decreased compared to modafinil (**4a**, **4b** and **4g**: P < 0.001, **#**5: P < 0.05, respectively).

**Figure 3:**
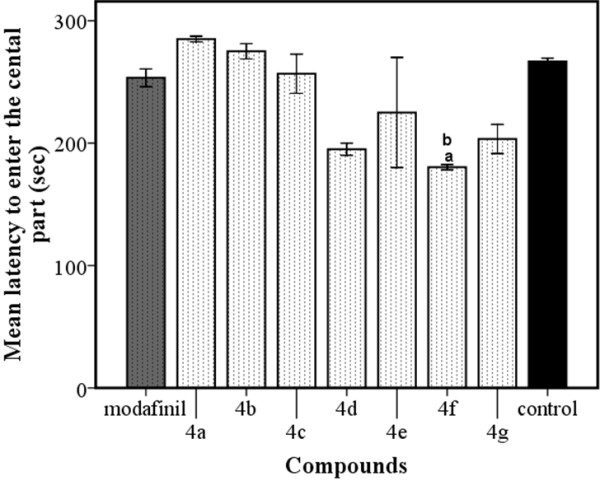
**Effects of different derivatives in open field test in mice.** Data are expressed as mean ± SEM of the latency to enter the central part. ^a^P < 0.05 *vs.* control, ^b^P < 0.05 *vs.* modafinil.

The results in Figure 3 showed that all compounds except compound **4f** failed to reach the statistically significant level in measuring the latency to enter the central part compared to control and modafinil groups. Compound **4f** showed had a reliable decrease in the latency to enter the central part compared to the both groups (P < 0.05).

#### FST

**Figure 4:**
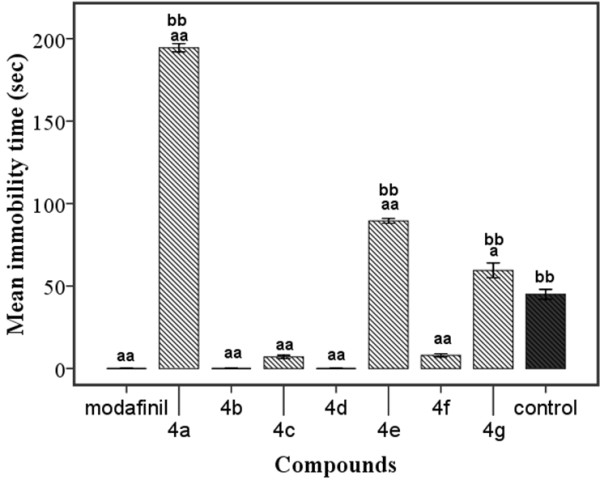
**Effect of different derivatives of modafinil on the immobility response in forced swimming test (**^**a**^**P < .05, **^**aa**^**P < .001 *vs. *** **control and **^**bb**^**P < 0.001 *vs. *** **modafinil groups).**

The effects of derivatives and modafinil on IT during trials were depicted in Figure 4. The control animals showed 45 ± 3 sec immobility duration during FST. Compounds **4a** and **4e** increased IT and showed considerable differences when compared to the respective control groups (P < 0.001). Compound **4g** also lengthened IT in comparison to control (P < 0.05). In addition, compounds **4a**, **4e**, **4g** and control showed a significant increase in IT compared to modafinil group (P < 0.001).

The rest of the compounds significantly decreased IT in comparison to control (P < 0.001).

#### EPM

Mice exposed to compounds **4a**, **4c**, **4e** and **4g** showed decline in open arms entries and open arms spent time in comparison to control mice (P < 0.05). However, modafinil, compounds **4b**, **4d** and control (DMSO) showed similar results (P > 0.05). (See Table 1.)

The percent of close arms entries revealed that all derivatives showed a non-significantly decrease in this parameter compared to those of control and modafinil groups.

Compound **4a**, **4c**, **4e** and **4g** produced a reliable increase of close time spend (P < 0.05) than control group.

## Conclusions

We have described a novel series of modafinil analogs (**4a-4g**) that displayed some kind of CNS activities. From our psychobiological results, compounds **4a**, **4c**, **4e** and **4g** decreased frequencies of open arms entries and duration of open arms spent times, suggesting an anxiogenic-like effect and as well as these derivatives increased close arms time significantly. Compounds **4a**, **4e** and **4g** also lengthened IT in FST, indicating that the derivatives exerted a depressive action, while other derivatives shortened IT and would be considered to have antidepressant effects. The square crossing numbers of compounds **4a**, **4b** and **4g** showed a significant reduction compared to modafinil and control groups which suggest this compound may be putative sedative. Compound **4f** induced an anxiolytic-like effect because it decreased the latency to enter the central part compared to other derivatives. The results of EPM also roughly confirmed the anxiolytic-like effect of compound **4f**. Based on LORR test, it is evident that most of the analogs exhibited stimulant activity in LORR test and compound **4c** is the most potent ones. Only compound **4a** (aniline substitution) was recognized as sedative analog. Finally, little discrepancies among results obtained from different psychobiological tests in this study may be related to the different mechanisms of actions of these derivatives and future studies are highly requested to exploit the structure-function relationships of these derivatives in more details.

## Competing interests

The authors declare that they have no competing interests.

## Authors’ contributions

AL: Synthesis of target compounds and performing the biological tests. IK: supervision of the psychobiological part. HA: collaboration in identifying of the structures of target compounds and manuscript preparation. AA: collaboration in identification of synthesized compounds. LF: collaboration in synthesis of target compound and manuscript preparation. AF: Design of target compounds and supervision of the synthetic part. All authors read and approved the final manuscript.
